# Tracheal myoepithelioma resected by using flexible video bronchoscope: a case report and review of the literature

**DOI:** 10.3389/fonc.2025.1709584

**Published:** 2026-01-06

**Authors:** Xiaosheng Meng, Jiguang Meng, Yaping Cao

**Affiliations:** Fourth Medical Center of People’s Liberation Army (PLA) General Hospital, Beijing, China

**Keywords:** flexible video bronchoscopy, myoepithelioma, salivary gland tumor, thoracic tumor, tracheal tumor

## Abstract

**Background:**

Tracheal myoepithelioma is a rare, low-grade malignant tumor originating from salivary gland tissue. This article presents a case of tracheal myoepithelioma without clinical symptoms, diagnosed and treated using flexible video bronchoscopy.

**Case Report:**

A 44-year-old male patient, asymptomatic for respiratory issues, was found to have a tracheal mass during a routine chest CT scan. The mass showed no significant changes over the course of one year. Upon admission, flexible video bronchoscopy revealed a coral-like neoplasm. The tumor was resected using a high-frequency electrocautery loop, followed by cryotherapy to remove residual tissue. Postoperative pathological examination confirmed the diagnosis of myoepithelioma. Three months later, follow-up flexible video bronchoscopy demonstrated good mucosal healing at the original tumor site, and chest CT showed no evidence of infiltration. The patient reported no discomfort during this period.

**Conclusion:**

Although tracheal myoepitheliomas are rare, they should be considered in the differential diagnosis of tracheal masses. Minimally invasive interventional therapy via flexible video bronchoscopy may serve as a valuable diagnostic and therapeutic approach.

## Introduction

Salivary gland tumors arise from the submucosal minor salivary glands and represent a rare tumor type (1). Myoepithelioma, a subtype of salivary gland tumor, accounts for approximately 1%–2% of all salivary gland tumors (2). It is most commonly found in the parotid gland, breast, lacrimal gland, and skin (3), with rare cases reported in the mediastinum and nasal cavity (4, 5). Myoepithelioma typically lacks specific clinical manifestations, and diagnosis relies primarily on pathological examination, which remains the gold standard. Current literature mainly consists of case series, with most patients showing no evidence of tumor recurrence. However, myoepitheliomas can exhibit a spectrum of behavior ranging from low-grade malignancy to highly malignant phenotypes, including locally invasive growth affecting surrounding fat, muscle, or nerves. Notably, an increased number of cases have been reported in pediatric patients (4).

Tracheal tumors are rare, low-grade malignant neoplasms of the respiratory tract. They have a low incidence and present with non-specific clinical symptoms, commonly including dyspnea and wheezing, which often leads to misdiagnosis as other respiratory conditions. Tracheal myoepithelioma is even rarer, with fewer than ten cases reported worldwide since the first description in 2011 (6), Most documented cases have been diagnosed and managed via bronchoscopy, with few instances of postoperative recurrence. This article describes a case of tracheal myoepithelioma diagnosed and treated using flexible video bronchoscopy.

## Case report

**Figure 1:**
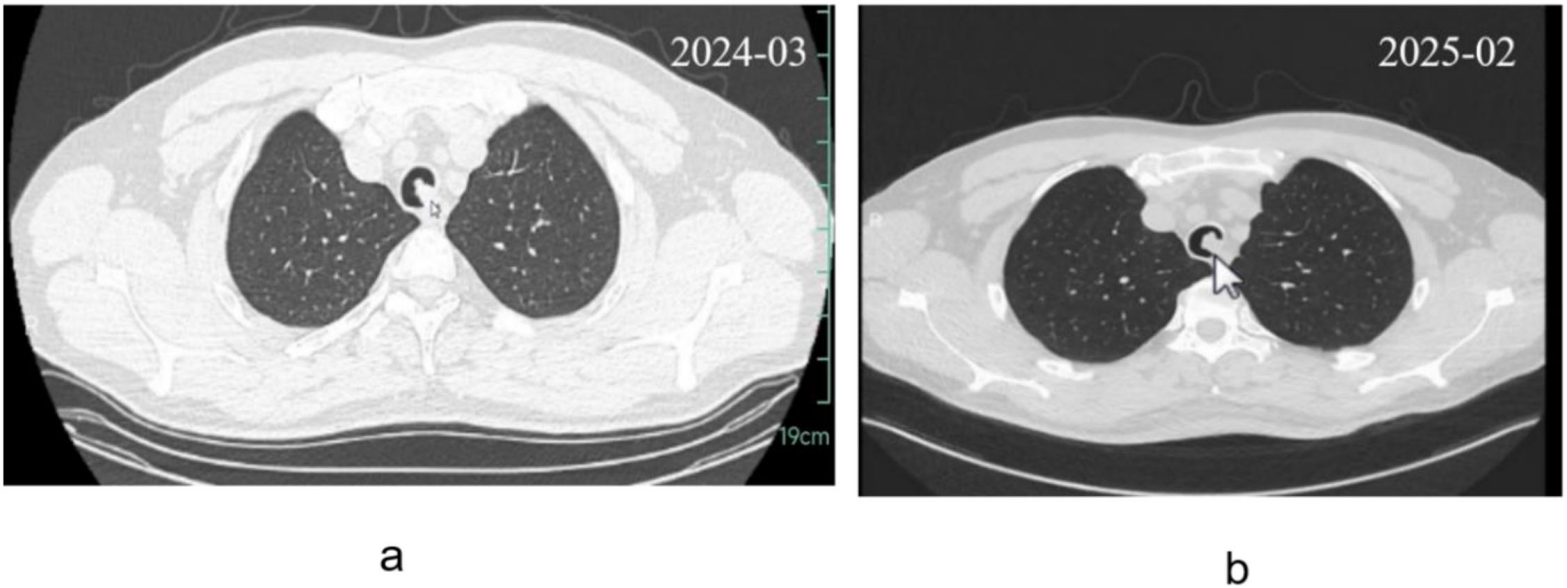
Chest CT shows the tracheal mass: **(a)** Initial imaging of the tracheal mass; **(b)** Follow-up imaging one year after discovering a tracheal mass.

The patient is a 44-year-old male with a history of occasional smoking. He is employed as a police officer and is regularly exposed to secondhand smoke. His general health condition is good, with no family history of genetic diseases. During a routine physical examination, a chest CT scan revealed an airway mass. Due to occupational demands and the absence of respiratory symptoms, no immediate treatment was initiated. The mass remained stable over the following year, prompting the patient to seek further evaluation at our hospital. From symptom onset to presentation, the patient remained asymptomatic. Chest CT images from physical examinations conducted in March 2024 and February 2025 were provided upon admission (**Figure 1**).

### Clinical differential diagnosis

In this case, imaging over a one-year period showed no significant changes, suggesting a benign etiology such as hamartomas or squamous cell papillomas, which are typically asymptomatic or cause only mild symptoms. These lesions often present as intraluminal nodules with associated tracheal wall thickening. However, given the patient’s smoking history, malignant conditions such as salivary gland-type tumors or squamous cell carcinoma should also be considered (1).

**Figure 2:**
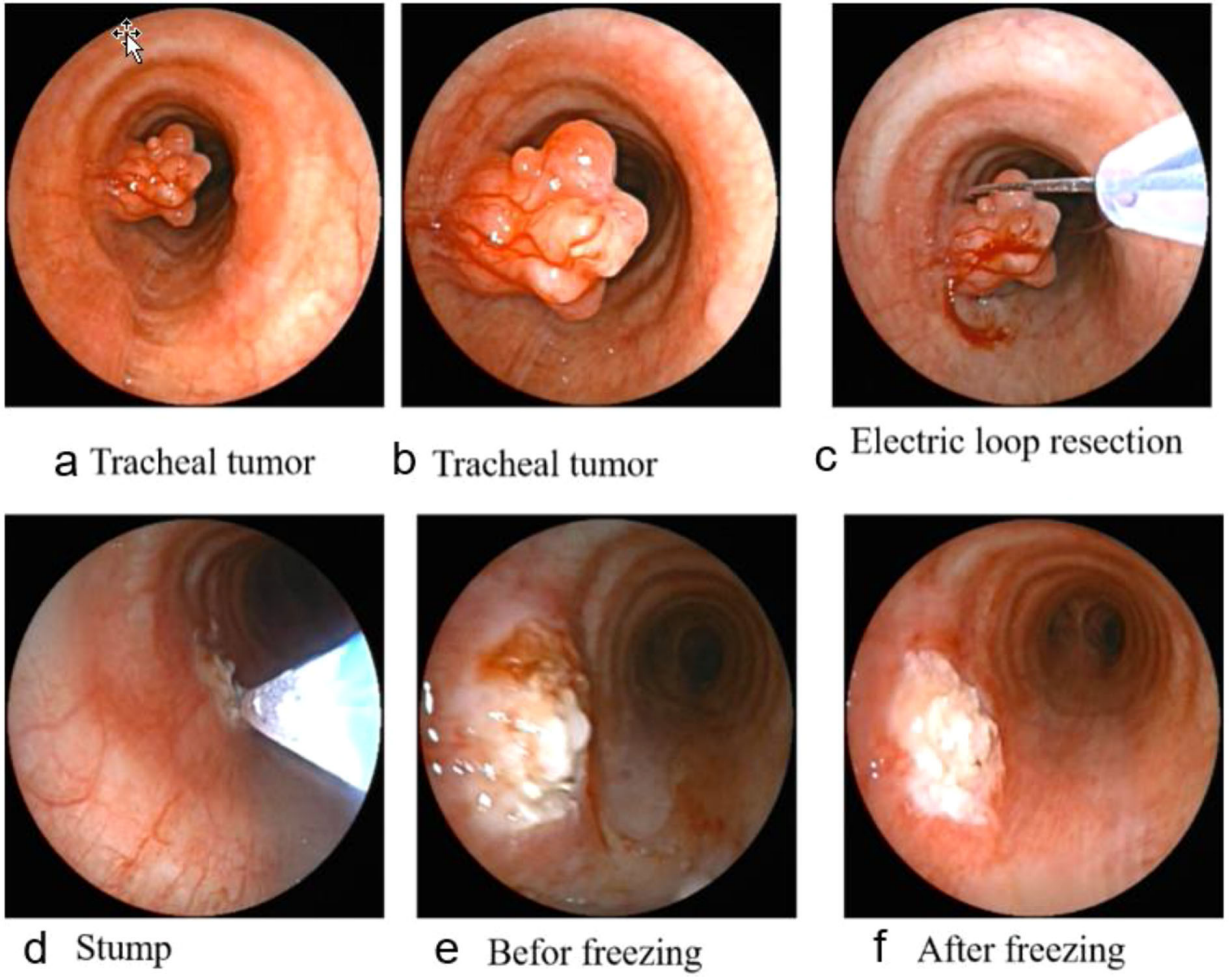
Under flexible video bronchoscopy: **(a b)** Morphology of tracheal mass; **(c)** Tracheal tumor resection with electrocautery snare; **(d)** Freeze-thaw by using a cryoprobe; **(e, f)** Before and after comparison of the same area after freezing.

Therefore, tumor markers were tested, and a PET-CT scan was performed. Upon admission, routine blood tests, tumor markers (including CA-125, CEA, CA19-9, NSE, and SCC), biochemical tests, and blood gas analysis showed no significant abnormalities. The subsequent PET-CT examination indicated that the tracheal mass was likely benign, with a maximum standardized uptake value (SUV max) of 3, and no evidence of distant metastasis was detected. Under general anesthesia, a laryngeal mask airway was inserted, and a flexible video bronchoscope was advanced through it. Endoscopic examination revealed a coral-like polyp protruding into the lumen on the left posterior wall of the trachea, approximately 6 cm from the carina. The lesion was firm and measured approximately 1.5 cm in maximum diameter (**Figure 2**). The polyp was resected using high-frequency electrocautery loop resection, followed by removal of residual tissue with a cryoprobe. The excised polyp was retrieved, and the remaining stump at the resection site was treated with six cycles of cryotherapy (freezing and thawing) using the cryoprobe. No bleeding occurred during or after the procedure.

**Figure 3:**
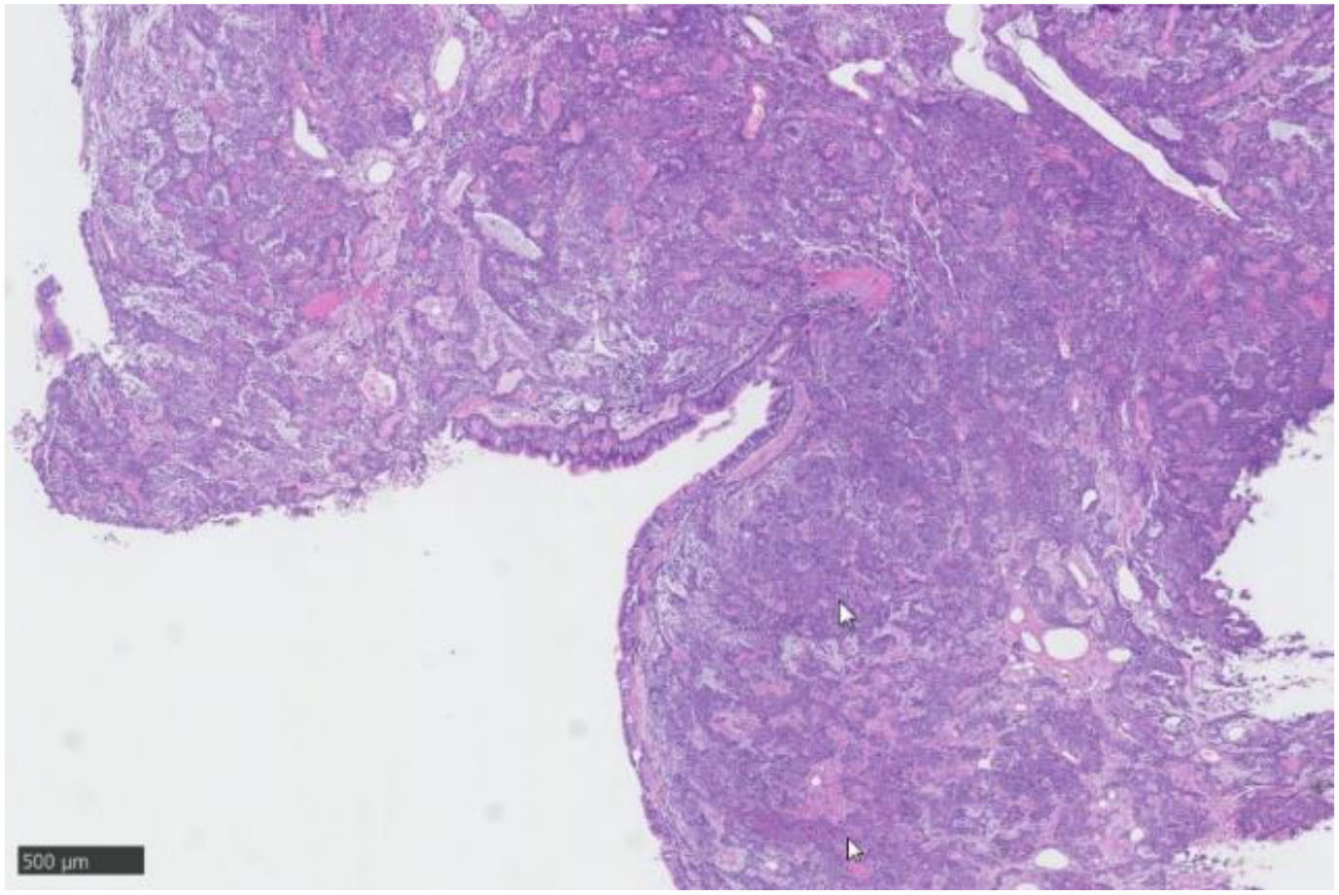
Pathology: Plasma cell-like cells can be seen at the site indicated by the arrow.

Pathology and immunohistochemistry: Bronchial salivary gland tumor, showing plasma cell-like cells (**Figure 3**); immunohistochemistry: CK7(++), CK20(-), P63(myoepithelial+), P40(++), Ki67(5%+), TTF(+), EMA(+), CD117(++), S100(++), CK5(++), CD56(+), Syn(-), CgA(-), CEA(-).

### Postoperative follow-up

**Figure 4:**
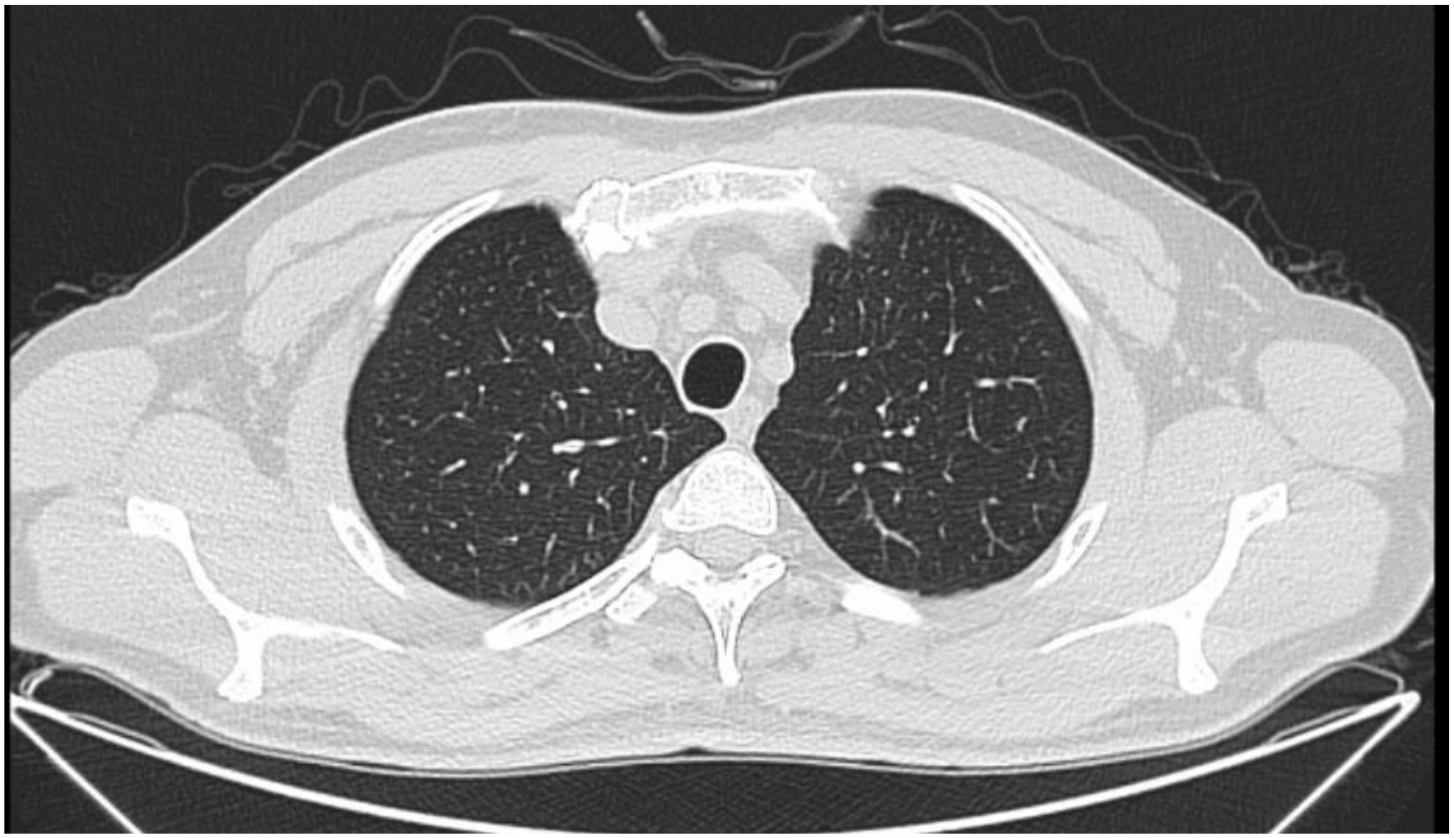
Postoperative Chest CT Scan: Chest CT imagine three months after removal of tracheal tumor: no significant thickening or infiltration of the trachea.

**Figure 5:**
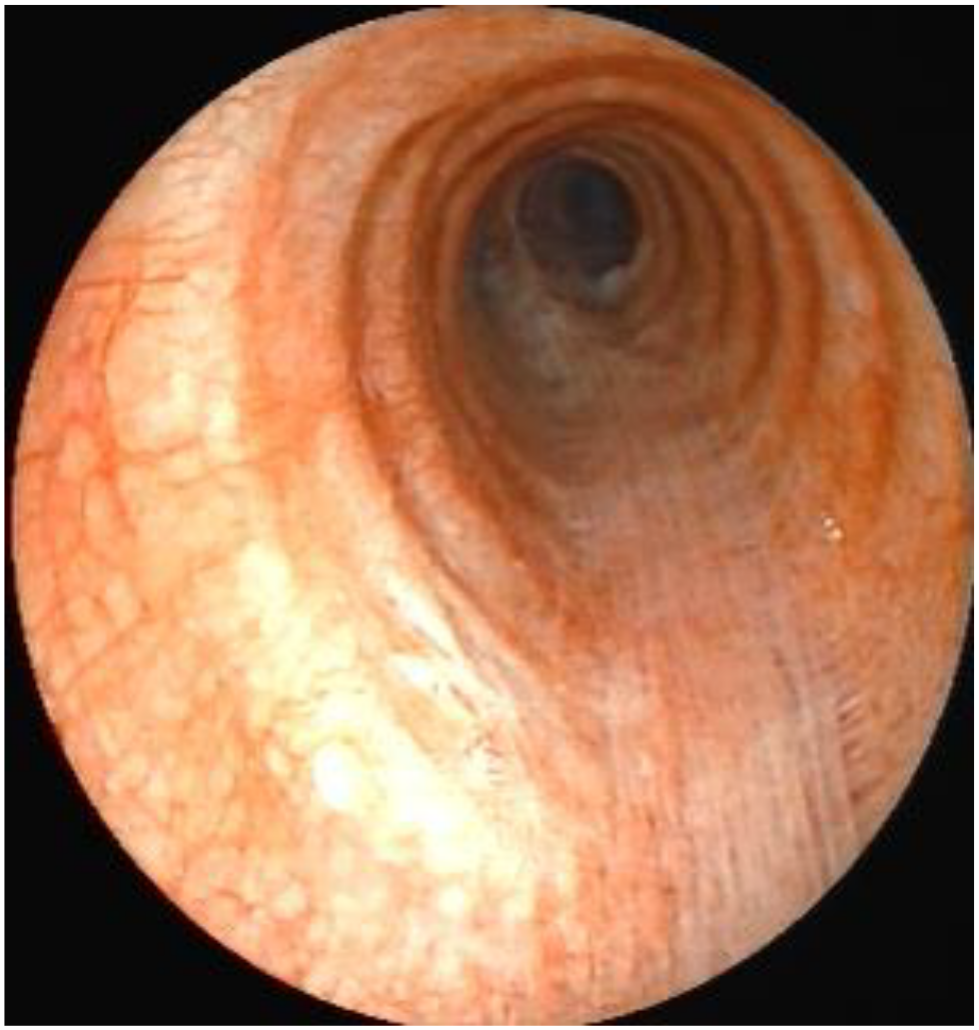
Postoperative stump: The flexible video bronchoscopy three months after removal of tracheal tumor: the resection site was well covered with mucosa.

Three months later, the patient returned for a follow-up examination. Chest CT revealed no significant thickening or infiltration of the trachea (**Figure 4**). Repeat flexible video bronchoscopy showed that the resection site was well covered with mucosa, with no evidence of recurrence and only slight scar hyperplasia (**Figure 5**). The patient remains under ongoing follow-up. We recommended a chest CT scan at six months post-surgery and a repeat flexible video bronchoscopy at nine months. However, since the patient reported no significant discomfort, the six-month chest CT was not performed **Table 1**.

**Table 1:** Summary of the entire clinical timeline.

Time	Event category		Patient initiative
2024-3	First discovery	Chest CT imaging shows a tracheal mass	Untreated
2025-2	Follow-up visit	Chest CT still shows a tracheal mass	Received medical treatment at our hospital
2025-3	Hospital Admission	Tracheal tumor resection under flexible video bronchoscopy	
2025-6	Hospital follow-up	No recurrence was observed on chest CT and flexible video bronchoscopy	
2025-9	Follow-up and re-examination		The patient did not return for follow-up

Summary of the entire clinical timeline.

## Discussion

Myoepithelioma is a rare salivary gland tumor first described by F. Tavassoli and colleagues in 1991. These tumors typically arise in tissues with endocrine functions, including the salivary glands, lacrimal glands, sweat glands, nasopharynx, and mammary lungs. The incidence of primary pulmonary myoepithelial tumors is higher than that of tracheal and bronchial myoepitheliomas. Diagnosis can be confirmed via biopsy, although most reported cases have been definitively diagnosed through surgical resection. Surgical resection currently represents the optimal treatment for primary pulmonary myoepithelioma. The pleomorphic appearance of myoepithelioma under electron microscopy is mainly due to the diversity of myoepithelial cells and the surrounding stroma, with four primary cell types identified: myoepithelial cells, plasma cell-like cells, spindle cells, and clear cells (7). Immunohistochemical staining typically demonstrates varying degrees of positivity for markers including smooth muscle actin (SMA), calmodulin, S-100 protein, cytokeratin (CK), and vimentin (**Table 2**). Combined immunohistochemical testing using multiple markers is considered to improve diagnostic accuracy for myoepitheliomas, with some studies suggesting that SMA and S-100 are the most valuable indicators (8). A recent study (9) demonstrated the utility of second-generation metagenomic sequencing in detecting pulmonary myoepithelioma, reporting no mutations, translocations, or amplifications in 56 genes commonly associated with thoracic tumors, including EGFR, ALK, ERBB2, MET, ROS1, and other proto-oncogenes and tumor suppressor genes. Imaging studies (10) are useful for confirming tumor location. Due to their diverse growth patterns, myoepitheliomas exhibit variable imaging features, including narrow-based intraluminal nodules, wide-based intraluminal nodules, and extraluminal mass-type lesions.

**Table 2:** Seven cases of tracheal myoepithelioma.

Reference	Sex	Age	Clinical manifestations	Smoking	Size	Follow-up
Sekine,et al,2014 (14)	Female	51	Coughing, expectoration, shortness of breath			No recurrence
Li Qiong, et al,2011 (13)	Female	43	Coughing, shortness of breath	No	1*1cm	
Li, Y., et al,2012 (6)	Male	23	Coughing, shortness of breath			No recurrence
Sekine, A., et al,2014 (15)	Female	67	Shortness of breath	No		No recurrence
Chand, M., et al,2011 (16)	Male	77	Coughing, expectoration, hemoptysis	Yes	0.7cm	
Pfeiffer,M., et al,2018 (8)	Female	10	Shortness of breath	No	1.4*1.9	Recurrence
Mardani,P, et al,2022 (17)	Male	36	Shortness of breath, chest pain, hemoptysis		2*2	No recurrence

Clinical characteristics of 7 Cases.

A literature review identified several reported cases of tracheal myoepithelioma (**Table 2**) and immunohistochemical markers (**Table 3**).

**Table 3:** Immunohistochemical markers of 7 case.

Reference		CK	S-100	Calponin	P63	SMA	Ki67	Vinmentin
Sekine, et al, 2014 (14)	(15)	++	+		+	+	+	+
Li Qiong, et al,2011 (13)	(10)	+	1+	+	+	+	1%	
Li, Y., et al, 2012 (6)	(6)	+		+	+			+
Sekine, A., et al, 2014 (15)	(16)				+	+		
Chand, M., et al, 2011 (16)	(17)	+	+	+	+	+		+
Pfeiffer, M., et al, 2018 (8)	(7)	+	+			+		
Mardani, P, et al, 2022 (17)	(18)	+			+	+		

Immunohistochemical markers of 7 Cases.

The tracheal myoepithelioma reported in this study was pathologically confirmed following loop resection via flexible video bronchoscopy. In the present case, the lesion exhibited a relatively low maximum standardized uptake value (SUVmax3) on PET-CT, consistent with benign tumors. However, PET-CT has limited accuracy in distinguishing low-grade malignant tumors from benign lesions. Therefore, definitive diagnosis of myoepithelioma relies on pathological examination. When a lesion appears morphologically suspicious on PET-CT but demonstrates low metabolic activity, myoepithelioma should be considered in the differential diagnosis (11) Although airway myoepitheliomas have been documented previously, the limited number of cases prevents clear conclusions regarding correlations between incidence and factors such as gender, age, or smoking history.

Surgical excision remains the primary treatment for myoepithelioma, with most patients experiencing favorable outcomes and very low recurrence rates. For intraluminal myoepitheliomas, interventional treatment via flexible video bronchoscopy may become the preferred approach. As reported in cases by Mardani, P. and colleagues, rigid bronchoscopy resection of tracheal myoepitheliomas results in less tissue damage compared to traditional surgery, with no recurrences observed during follow-up.

Primary salivary gland tumors of the lung are relatively rare and are associated with a low diagnostic yield from transbronchial biopsy, often necessitating surgical resection for definitive diagnosis. In contrast, tracheal myoepitheliomas are more commonly diagnosed and treated via transbronchial resection—a minimally invasive endoscopic intervention—particularly in cases presenting with severe obstruction and respiratory distress. Although recurrence of tracheal myoepithelioma is rare, multiple recurrences following several endoscopic resections have been reported (12). In such cases, tracheal resection followed by end-to-end anastomosis has been successful in preventing further recurrence. Case reports (13) have indicated that pulmonary myoepitheliomas can infiltrate adjacent lung tissue and hilar lymph nodes and may metastasize to the contralateral lung after surgical resection. Additionally, metastases to distant sites such as the liver and hip have also been documented (14).

Currently, there is no clear evidence supporting the need for postoperative adjuvant therapy in cases of tracheal myoepitheliomas. Previous reports have described various methods for removing tracheal masses via flexible video bronchoscopy, including rigid bronchoscopy, flexible video bronchoscopic resection, electrocoagulation, and argon plasma coagulation. All these techniques have demonstrated favorable outcomes, with complete tumor resection achieving excellent hemostasis and low recurrence rates. Therefore, flexible video bronchoscopic resection (minimally invasive bronchoscopic therapy) remains the primary approach for symptom relief and disease diagnosis.

The patient continues to be monitored and underwent a repeat bronchoscopy three months postoperatively for evaluation. No recurrence was observed at the original tumor site; only minimal scar tissue was present, covering the stump with well-healed tracheal mucosa. To assess for peripheral tracheal growth, a follow-up chest CT revealed thinning of the tracheal wall at the original tumor site without evidence of infiltrative growth. These findings align with previously reported follow-up outcomes for tracheal myoepitheliomas. Although recurrence and metastasis rates for tracheal myoepitheliomas are low, regular follow-up examinations remain essential to evaluate both the postoperative tracheal stump and the tracheal lumen. During follow-up, the patient avoided exposure to secondhand smoke and reported no impact on daily activities or exercise routines. While the patient did not comply with the medical recommendation for a follow-up chest CT scan, they expressed willingness to undergo another bronchoscopy six months post-surgery to assess the status of the primary lesion.

## Conclusion

An increasing number of studies have reported airway-associated myoepitheliomas, with tracheal myoepitheliomas particularly prone to causing airway obstruction. Risk assessment based on clinical presentation, patient age, and lesion size is essential to determine the need for further treatment. While surgical resection remains the gold standard, current management increasingly favors minimally invasive techniques such as transbronchial loop resection, high-frequency electrocautery, and cryotherapy, which result in significantly less trauma compared to surgery. Myoepitheliomas carry a risk of recurrence; if recurrence occurs during follow-up, end-to-end anastomosis following tracheal resection may be considered. In summary, although tracheal myoepitheliomas are rare tumors with a low rate of metastasis, minimally invasive treatments via flexible video bronchoscope can serve as the preferred initial option after appropriate evaluation.

## Data Availability

The original contributions presented in the study are included in the article/supplementary material. Further inquiries can be directed to the corresponding author.
